# Impact of weight change during neoadjuvant chemotherapy on pathologic response in triple-negative breast cancer

**DOI:** 10.1002/cam4.388

**Published:** 2015-01-30

**Authors:** Jean Bao, Nicholas Borja, Madhu Rao, James Huth, A Marilyn Leitch, Aeisha Rivers, Rachel Wooldridge, Roshni Rao

**Affiliations:** Department of Surgery, Division of Surgical Oncology, University of Texas Southwestern Medical Center5323 Harry Hines Blvd., Dallas, Texas, 75390-9155

**Keywords:** Ki-67, neoadjuvant chemotherapy, triple-negative breast cancer, weight change

## Abstract

Triple-negative breast cancer (TNBC) is an uncommon but aggressive subtype of breast cancer. Obesity has been associated with an increased risk of breast cancer and worse prognosis. Some studies suggest that obese patients are less likely to achieve pathologic complete response (pCR) to neoadjuvant chemotherapy (NCT) and experience worse overall survival. Ki-67 is a proliferation marker that correlates with tumor aggressiveness. The goal of this study was to examine the impact of weight change during NCT for TNBC on pathologic response and Ki-67 reduction. Retrospective review identified 173 TNBC patients treated between 2004 and 2011. Data were collected on patient demographics, pre- and post-NCT body mass index (BMI), Ki-67, and pCR. Data analysis was performed using the two-tailed Student's *t*-test, analysis of variance (ANOVA), and Fisher's exact test. Sixty-six patients met final study criteria. Forty-three patients lost weight during chemotherapy and 23 gained weight. Patients in the weight gain group were significantly younger (*P* = 0.0013). There was no significant difference between the two groups in terms of Ki-67 reduction (*P* = 0.98) or pCR (*P* = 0.58). When patients were separated into normal weight (BMI<25 kg/m^2^), overweight (BMI ≥ 25 and <30 kg/m^2^), and obese (BMI ≥ 30 kg/m^2^), there was no significant difference in Ki-67 among those groups either before or after NCT. The degree of obesity did not have a significant impact on Ki-67 reduction. Weight change during NCT does not appear to correlate with Ki-67 change or achieving pCR in TNBC. This may reflect the nature of this subtype of breast cancer that is less responsive to the hormonal effects that adipose tissue exerts on cancer cell proliferation.

## Introduction

Triple-negative breast cancer (TNBC) is characterized by the absence of estrogen, progesterone, and human epidermal growth factor receptor 2 (HER2) receptors. It represents a distinct subtype of breast cancer found in 15–20% of invasive breast cancers diagnosed in the United States. It is more common in African–Americans and in premenopausal women 1. Despite a lower prevalence rate than hormone receptor-positive breast cancers, TNBC leads to a disproportionate number of breast cancer deaths and imparts a significantly lower survival rate, likely due to the aggressive nature of the disease and a lack of targeted therapeutic options 1,2. Chemotherapy remains the mainstay therapy for TNBC.

Obesity, as measured using body mass index (BMI), has generally been associated with both an increased risk of breast cancer as well as an overall poorer prognosis after diagnosis 3–5. There is increasing evidence that weight change during chemotherapy is associated with decreased survival and increased recurrence risk 6–9. Even though the underlying mechanism remains unclear, it has been proposed that the increased circulating levels of estrogen, insulin, insulin-like growth factor (IGF), and other hormonal factors in obese patients may promote proliferation of breast cancer cells 10–12.

Ki-67, originally discovered at Kiel University in Germany, is a tumor marker commonly obtained on breast tumors. A nuclear protein associated with cellular proliferation has been shown to be an independent prognostic marker for disease-free and overall survival 13–15. Additionally, a lower Ki-67 after neoadjuvant chemotherapy (NCT) is associated with improved disease-specific survival and lower recurrence rates 15,16.

NCT for breast cancer provides a unique opportunity to evaluate intervention efforts, including response to chemotherapeutic agents and lifestyle interventions. Some studies suggest that obese patients are less likely to have pathologic complete response (pCR) to NCT and are more likely to experience worse overall survival 7,8. This may be partly due to the finding that obese patients present with larger and more clinically advanced tumors, and may also be attributed to the potential under dosing of chemotherapy in obese patients resulting in diminished clinical effectiveness. A prospective, randomized pilot trial conducted at our institution randomized women undergoing NCT for estrogen receptor-positive (ER+) breast cancer to a supervised exercise program and to a control group 17. Patients in the exercise program had a significantly lower BMI after NCT and had significantly greater reductions in Ki-67 compared to controls.

Given the obesity epidemic in the United States and its adverse association with breast cancer outcomes, especially in the setting of a disease that is aggressive and lacks effective targeted therapeutic options, we sought to examine the impact of weight change during NCT for TNBC on Ki-67 and pathologic response.

## Methods

This study was approved by the Institutional Review Board of the University of Texas Southwestern (UTSW) Medical Center. Parkland Memorial Hospital (PMH) is a Dallas county hospital that serves a primarily indigent population. Harold C. Simmons Cancer Center (SCC) is a National Cancer Institute's designated cancer center that serves primarily insured patients. Both PMH and SCC are affiliated with UTSW Medical Center and are accredited members of the Commission on Cancer. Both PMH and SCC are staffed by the same clinical faculty, residents, and medical students. Clinical protocols at both centers are identical. Approximately 500 breast cancers are treated annually at both centers.

Tumor registry data were queried to identify all patients diagnosed with TNBC between 2004 and 2011 at PMH and SCC. Information was collected on demographics, tumor size, types of treatment, and therapeutic outcome. Additionally, weight and Ki-67 data were collected for pre- and post-NCT, while pCR status was collected post-NCT. Those without complete data on weight, height, or Ki-67 were excluded.

Weight classes are categorized based on BMI, which is calculated by dividing weight (kg) by height (m) squared: normal weight 18.5–24.9 kg/m^2^, overweight 25–29.9 kg/m^2^, obese 30–34.9 kg/m^2^, severely obese 35–39.9 kg/m^2^, morbidly obese ≥40 kg/m^2^. Initial BMI was most often recorded at the first visit with medical oncology to discuss NCT. Final BMI was evaluated at the time of preoperative anesthesia evaluation. Due to the weight-based administration of NCT and anesthesia, these BMIs were the most accurately and consistently recorded at our institution.

Breast cancer diagnosis was made by core needle biopsy. Immunohistochemistry (IHC) was used to determine hormone receptor status. HER2/neu status was evaluated by IHC or by fluorescent in situ hybridization. Ki-67 was measured using quantitative IHC in the initial core biopsy specimen and then again in the final resection specimen removed for definitive surgical intervention after NCT.

Statistical analysis was performed using a two-tailed Student's *t*-test, analysis of variance (ANOVA), and Fisher's exact test. A *P *< 0.05 was considered statistically significant. Analyses were conducted using Graphpad Prism.

## Results

One hundred and seventy-three TNBC patients were identified. Sixty-six patients had complete data and were included in the final analysis. Twenty-three patients gained weight during NCT with an average BMI gain of 1.4 kg/m^2^, and 43 patients lost weight with an average BMI reduction of 2.1 kg/m^2^ (Table1). The difference in BMI change between the two groups was significant (*P* < 0.0001). The average length of time between pre-NCT and post-NCT measurements was 138 days with a range of 77–210 days.

**Table 1:** Mean BMI and BMI change in weight loss and weight gain groups (BMI in kg/m^2^)

	All (*n* = 66)	Weight gain (*n* = 23)	Weight loss (*n* = 43)	*P* value^1^
Initial BMI	31.7 ± 9.6	30.4 ± 7.1	32.4 ± 10.6	0.4
Final BMI	30.8 ± 9.2	31.8 ± 6.9	30.3 ± 10.2	0.5
BMI change	−0.9 ± 2.6	1.4 ± 0.3	−2.1 ± 0.3	<0.0001
*P* value^2^	0.60	0.48	0.35	

BMI, body mass index.

1 Comparing weight gain and weight loss group.

2 Comparing initial BMI and final BMI.

Table2 describes the overall patient population. The weight gain group was significantly younger than the weight loss group (44 vs. 53 years, *P* = 0.0013). The average initial BMI was 31.7. Almost half of the patients were obese (47%), and 74% were either overweight or obese.

**Table 2:** Patient characteristics

	All (*n* = 66)	Weight gain (*n* = 23)	Weight loss (*n* = 43)	*P* value
Age (years)	50.0 (26–75)^1^	44.4 ± 2.0	53.0 ± 1.5	0.0013
Race				
African–American	28 (42%)	5 (22%)	23 (53%)	0.02^2^
Hispanic	19 (29%)	10 (43%)	9 (21%)	0.08^3^
White	16 (24%)	6 (26%)	10 (23%)	
Other	3 (5%)	2 (9%)	1 (2%)	
Initial BMI (kg/m^2^)	31.7 (18.6–70)^1^	30.4 ± 7.1	32.4 ± 10.6	0.4
Cancer type				
IDC	64 (97%)	22 (96%)	42 (98%)	
Poorly differentiated	2 (3%)	1 (4%)	1 (2%)	
Initial tumor size (cm)	5.6 (0–21)	4.9 ± 4.2	6.0 ± 4.0	0.25
Initial Ki-67	74.5 (4–100)^1^	72.9 ± 20.1	75.4 ± 23.7	0.66
Chemotherapy				
TAC	54 (82%)	18 (78%)	36 (84%)	0.35
Platinum-based	10 (15%)	4 (17%)	6 (14%)	
Other	2 (3%)	1 (4%)	1 (2%)	
Breast surgery				
TM	31 (47%)	11 (48%)	20 (47%)	1.0
PM	34 (52%)	12 (52%)	22 (51%)	
None	1 (1.5%)		1 (2%)	
Axillary surgery				
SLNB	20 (30%)	7 (30%)	13 (30%)	1.0
ALND	41 (62%)	15 (65%)	26 (60%)	
SLNB→ALND	4 (6%)	1 (4%)	3 (7%)	
None	1 (1.5%)		1 (2%)	

IDC, invasive ductal carcinoma; TAC, taxane, adriamycin, cyclophosphamide; TM, total mastectomy; PM, partial mastectomy; SLNB, sentinel lymph node biopsy; ALND, axillary lymph node dissection.

1 Mean (range).

2 African–American versus all.

3 Hispanic versus all.

African–Americans accounted for 42% of the included TNBC patients. In the weight loss group, there were significantly more African–Americans than in the weight gain group (*P* = 0.02). However, the weight gain group had almost twice as many Hispanic patients as the weight loss group (*P* = 0.08).

The average initial tumor size was 4.7 cm and 6.0 cm for the weight gain group and for the weight loss group, respectively (Table3). After NCT, both groups had a reduction in tumor size, but the reduction was not significantly different between the two groups (*P* = 0.35). Both groups also had a reduction in Ki-67 after NCT, from 72.9% to 35.4% for the weight gain group, and from 75.4% to 37.8% for the weight loss group. The change in Ki-67 between the two groups was not significant (*P* = 0.98). The weight gain group had 9% more pCR patients than did the weight loss group, but the difference did not achieve statistical significance (*P* = 0.58).

**Table 3:** Change in Ki-67, tumor size, and pathologic complete response (pCR) in weight gain and weight loss groups

	All (*n* = 66)	Weight gain (*n* = 23)	Weight loss (*n* = 43)	*P* value
Ki-67				
Initial	74.5 ± 22.4	72.9 ± 20.1	75.4 ± 23.7	0.66
Final	36.9 ± 35.6	35.4 ± 33.2	37.8 ± 37.1	0.79
Reduction	37.6 ± 41.7	37.5 ± 44.2	37.7 ± 40.8	0.98
Tumor size (cm)				
Initial	5.6 ± 4.1	4.7 ± 4.2	6.0 ± 4.0	0.25
Final	2.0 ± 3.3	1.9 ± 2.2	2.1 ± 3.9	0.84
Reduction	3.5 ± 4.2	2.9 ± 3.7	3.9 ± 4.4	0.35
pCR	22 (33%)	9 (39%)	13 (30%)	0.58

The patients were divided into normal weight, overweight, and obese (*n* = 17, 18, 31). There was no significant difference in Ki-67 among the three groups either before NCT or after NCT. When the obese patients were further divided into obese, severely obese, and morbidly obese (*n* = 9, 15, 7 respectively), there was no significant different in Ki-67 reduction among those three groups (*P* = 0.16).

## Discussion

TNBC represents a distinctive and less common disease process that lacks effective targeted treatments. Compared to hormone receptor-positive tumors, these cancers have an earlier onset, are more likely to metastasize, and lead to a shorter survival. There are only a few studies examining the relationship between obesity and TNBC, mostly focusing on the incidence of TNBC 18–20. This study was the first to examine the impact of weight change during NCT on TNBC using outcome measures such as tumor size, Ki-67, and pCR, and found that weight change during NCT was not significantly associated with any of those measures for TNBC.

African–Americans comprised 42% of the TNBC patients in this study, almost twice as many as Whites. This is consistent with the literature finding that TNBC is significantly more prevalent in African–Americans, 26% versus 16% among non-African–Americans in the Carolina Study 1. The high proportion of African–Americans and Hispanics in this study (71%) may also reflect the patients served by PMH—a hospital that focuses on an indigent, uninsured population, and where 60% of the patients are Hispanics and African–Americans 21. In fact, 80% of the patients treated for breast cancer at PMH between 2005 and 2008 were minorities, the largest group being African–Americans 22.

The relationship between weight and the risk of TNBC is more controversial. Some suggest obesity was associated with an increased risk of TNBC among postmenopausal nonhormone therapy users 18,19, while others did not find such association 20. In fact, Stead et al. found that as BMI increased, the proportion of TNBC decreased. This is in contrast to the positive association between BMI and hormone receptor-positive breast tumors, which is well characterized in the literature 23–25.

Even though weight loss is a consequence in many cancers, weight gain is frequently reported in women receiving chemotherapy for breast cancer 26–29. The women who gained weight in this study were significantly younger than those who lost weight. This is consistent with the literature finding that weight gain is more pronounced among premenopausal women 6,30–32. A combination of factors may contribute to this finding, including the type of chemotherapy, length of treatment, as well as ovarian failure and behavioral and lifestyle differences.

There is increasing evidence that weight change during chemotherapy is associated with worse oncologic prognosis. Two studies specifically reviewing NCT in breast cancer patients indicate that obese patients are significantly less likely to achieve pCR and are more likely to experience worse overall survival 7,8. Rao et al. showed in their randomized study that for ER+ tumors, a reduction in BMI during NCT was associated with a reduction in Ki-67 17. This is in contrast to the results of this study which does not reveal a significant association between weight change during NCT and Ki-67 reduction or pCR for TNBC.

The difference may be due to the fact that TNBC lacks hormone receptors and are thus relatively insensitive to the hormonal effects that adipose tissue exerts on breast cancer cell proliferation. Estrogen is one of the factors implicated in the positive association between BMI and breast cancer risk, as there are higher circulating levels of estrogen in obese patients 11,12,33. Estradiol, the estrogen most strongly associated with breast cancer, is converted to estrone in the liver and to either 2- or 16*α*-hydroxyestrone (2-OHE1 or 16*α*-OHE1) in target cells. 16*α*-OHE1 binds estrogen receptor much more strongly than the other and retains potent hormonal activities. Laboratory and clinical data suggest that in nonhormone therapy users, greater BMI and higher levels of 16*α*-OHE1 jointly and individually associates with an increased risk of breast cancer 34. Such an association may be less evident in tumors without hormone receptors.

As almost half of the weight gain group consisted of Hispanics and the weight loss group of African–Americans, race may play a role in weight variation during chemotherapy. In terms of survival outcomes, BMI seems to play less of a determinate role among African–Americans than Whites (Caucasians). In the Cancer Prevention Study II that studied 21,142 African–American women and 409,093 white women, BMI was more strongly associated with the risk of fatal breast cancer in Whites than African–Americans 35. In a more recent study, the association between obesity and greater risk of all-cause mortality and breast cancer-specific mortality was observed among white women but not African–Americans 36. African–Americans are more likely than Whites to have ER tumors, more comorbities, lower education levels, and are more likely to present with advanced disease. However, it remains unclear why the obesity–mortality association is less evident among African–Americans than Whites. Other than socioeconomic, behavioral, and healthcare access factors, one possible explanation may be that African–Americans, who tend to be more overweight than Whites, are also more likely to have TNBC, which may not respond estrogen-mediated factors as hormone receptor-positive tumors do.

The change in Ki-67 was not significantly different among the three obesity groups in our study. However, the degree of obesity, especially before cancer diagnosis, may impact survival. In the After Breast Cancer Pooling Project, underweight and morbidly obese women before breast cancer diagnosis had the greatest risk of overall mortality and breast cancer-specific mortality, compared to normal weight. Severe obesity and obesity conferred a small but nonsignificant increase in risk, and overweight was not associated with excess risk compared to normal weight women 37. Additionally, weight loss >10% post diagnosis was associated with increased mortality compared to weight stable 30. Weight gain >10% post diagnosis was only marginally related to overall mortality 38. Prediagnosis BMI appeared to be a strong predictor of cancer prognosis, and weight gain after diagnosis did not confer significant additional risk. Perhaps more focus should be placed on weight maintenance and avoiding large weight shifts after diagnosis. However, a recent meta-analysis showed that higher BMI was associated with higher overall and breast cancer-specific mortality with a linear dose–response, regardless of when BMI was ascertained 39.

The vast majority of the patients in those studies had ER+ or PR+ tumors. Thus, its applicability to TNBC remains unclear. Furthermore, exercise status was not assessed in those studies or in ours. Exercise has been shown to conserve fat-free mass during weight loss by dieting 40. Cachexia-induced loss of fat-free mass during breast cancer treatments may be related to chronic inflammation, reduced physical activity, toxicity to chemotherapy, as well as underlying comorbidities. All of these factors may be reasons for reduced survival. Fat-free mass, similar to adipose tissue, may play a role in cancer proliferation through various endocrine and immunologic pathways. Randomized control trials are needed to assess interventions for weight loss or maintenance on breast cancer survival.

There are limitations to this study. The first is the small sample size, which limits the power of the study. Many TNBC patients in the registry did not have complete weight or height data and were thus excluded. Patients included, however, were all true TNBCs, based on receptors obtained on the core needle biopsy specimen and confirmed on the surgical excision. With improvement in the electronic medical record system, we expect to have more patients with complete data for future studies. We did not assess exercise, menopausal status, or usage of hormone therapy or steroids, all of which could impact weight change and breast cancer risk and survival. Race and socioeconomic factors should also be further studied. Our patient population had a high initial BMI, and weight variation may be less pronounced because of that than with a leaner population. Weight loss could also represent a regression to the mean given its higher starting BMI compared to the weight gain group, or it could be a reflection of tumor progression.

This is the first study to examine the impact of weight change during NCT for TNBC. The relationship between BMI and breast cancer is extremely complex, and the prognostic value of weight variation is something becoming increasingly studied. It is of particular interest in the setting of TNBC because of the rare but aggressive nature of the disease and the lack of effective therapeutic strategies. In this study, we did not find a significant association between weight change and Ki-67 reduction or pCR, possibly due to the hormone insensitivity inherent to this type of tumor. However, it provides a platform for further investigation into the relationship between weight and TNBC, in the hopes of improving the outcome of this disease.

## Conclusion

Weight change during NCT for TNBC did not change the rate of pCR, nor impact reduction in Ki-67 levels. Opportunities exist to stratify the response across socioeconomic, age, and race-based cohorts.

## Conflict of Interest

None declared.

## References

[b1] Carey LA, Perou CM, Livasy CA, Dressler L, Cowan D, Conway K (2006). Race, breast cancer subtypes, and survival in the Carolina Breast Cancer Study. JAMA.

[b2] Hudis CA, Gianni L (2011). Triple-negative breast cancer: an unmet medical need. Oncologist.

[b3] Kroenke CH, Chen WY, Rosner B, Holmes MD (2005). Weight, weight gain, and survival after breast cancer diagnosis. J. Clin. Oncol.

[b4] Loi S, Milne RL, Friedlander ML, McCredie M, Giles G, Hopper J (2005). Obesity and outcomes in premenopausal and postmenopausal breast cancer. Cancer Epidemiol. Biomarkers Prev.

[b5] Senie RT, Rosen PP, Rhodes P, Lesser ML, Kinne DW (1992). Obesity at diagnosis of breast carcinoma influences duration of disease-free survival. Ann. Intern. Med.

[b6] Camoriano JK, Loprinzi CL, Ingle JN, Therneau TM, Krook JE, Veeder MH (1990). Weight change in women treated with adjuvant therapy or observed following mastectomy for node-positive breast cancer. J. Clin. Oncol.

[b7] Chen S, Chen CM, Zhou Y, Zhou RJ, Yu KD, Shao ZM (2012). Obesity or overweight is associated with worse pathological response to neoadjuvant chemotherapy among Chinese women with breast cancer. PLoS ONE.

[b8] Litton JK, Gonzalez-Angulo AM, Warneke CL, Buzdar A, Kau S, Bondy M (2008). Relationship between obesity and pathologic response to neoadjuvant chemotherapy among women with operable breast cancer. J. Clin. Oncol.

[b9] Thivat E, Therondel S, Lapirot O, Abrial C, Gimbergues P, Gadea E (2010). Weight change during chemotherapy changes the prognosis in non metastatic breast cancer for the worse. BMC Cancer.

[b10] Gadea E, Thivat E, Planchat E, Morio B, Durando X (2012). Importance of metabolic changes induced by chemotherapy on prognosis of early-stage breast cancer patients: a review of potential mechanisms. Obes. Rev.

[b11] Lorincz AM, Sukumar S (2006). Molecular links between obesity and breast cancer. Endocr. Relat. Cancer.

[b12] Yager JD, Davidson NE (2006). Estrogen carcinogenesis in breast cancer. N. Engl. J. Med.

[b13] Guarneri V, Piacentini F, Ficarra G, Frassoldati A, D'Amico R, Giovannelli S (2009). A prognostic model based on nodal status and Ki-67 predicts the risk of recurrence and death in breast cancer patients with residual disease after preoperative chemotherapy. Ann. Oncol.

[b14] Inwald EC, Klinkhammer-Schalke M, Hofstadter F, Zeman F, Koller M, Gerstenhauer M (2013). Ki-67 is a prognostic parameter in breast cancer patients: results of a large population-based cohort of a cancer registry. Breast Cancer Res. Treat.

[b15] Nishimura R, Osako T, Okumura Y, Hayashi M, Arima N (2010). Clinical significance of Ki-67 in neoadjuvant chemotherapy for primary breast cancer as a predictor for chemosensitivity and for prognosis. Breast Cancer.

[b16] Billgren AM, Rutqvist LE, Tani E, Wilking N, Fornander T, Skoog L (1999). Proliferating fraction during neoadjuvant chemotherapy of primary breast cancer in relation to objective local response and relapse-free survival. Acta Oncol.

[b17] Rao R, Cruz V, Peng Y, Harker-Murray A, Haley B, Zhao H (2012). Bootcamp during neoadjuvant chemotherapy for breast cancer: a randomized pilot trial. Breast Cancer.

[b18] Phipps AI, Buist DS, Malone KE, Barlow W, Porter P, Kerlikowske K (2012). Breast density, body mass index, and risk of tumor marker-defined subtypes of breast cancer. Ann. Epidemiol.

[b19] Phipps AI, Malone KE, Porter PL, Daling JR, Li CI (2008). Body size and risk of luminal, HER2-overexpressing, and triple-negative breast cancer in postmenopausal women. Cancer Epidemiol. Biomarkers Prev.

[b20] Stead LA, Lash TL, Sobieraj JE, Chi D, Westrup J, Charlot M (2009). Triple-negative breast cancers are increased in black women regardless of age or body mass index. Breast Cancer Res.

[b21] Gofin J, Gofin R (2011). Essentials of global community health.

[b22] Brazda A, Estroff J, Euhus D, Leitch A, Huth J, Andrews V (2010). Delays in time to treatment and survival impact in breast cancer. Ann. Surg. Oncol.

[b23] Ahn J, Schatzkin A, Albanes JV, Lacey D, Ballard-Barbash R, Adams K (2007). Adiposity, adult weight change, and postmenopausal breast cancer risk. Arch. Intern. Med.

[b24] Suzuki R, Orsini N, Saji S, Key TJ, Wolk A (2009). Body weight and incidence of breast cancer defined by estrogen and progesterone receptor status–a meta-analysis. Int. J. Cancer.

[b25] Suzuki R, Rylander-Rudqvist T, Ye W, Saji S, Wolk A (2006). Body weight and postmenopausal breast cancer risk defined by estrogen and progesterone receptor status among Swedish women: a prospective cohort study. Int. J. Cancer.

[b26] Goodwin PJ, Ennis M, Pritchard KI, McCready D, Koo J, Sidlofsk S (1999). Adjuvant treatment and onset of menopause predict weight gain after breast cancer diagnosis. J. Clin. Oncol.

[b27] Levine EG, Raczynski JM, Carpenter JT (1991). Weight gain with breast cancer adjuvant treatment. Cancer.

[b28] Nissen MJ, Shapiro A, Swenson KK (2011). Changes in weight and body composition in women receiving chemotherapy for breast cancer. Clin. Breast Cancer.

[b29] Tredan O, Bajard A, Meunier A, Roux P, Fiorletta I, Gargi T (2010). Body weight change in women receiving adjuvant chemotherapy for breast cancer: a French prospective study. Clin. Nutr.

[b30] Caan BJ, Kwan ML, Hartzell G, Castillo A, Slattery M, Sternfeld B (2008). Pre-diagnosis body mass index, post-diagnosis weight change, and prognosis among women with early stage breast cancer. Cancer Causes Control.

[b31] Demark-Wahnefried W, Rimer BK, Winer EP (1997). Weight gain in women diagnosed with breast cancer. J. Am. Diet. Assoc.

[b32] Demark-Wahnefried W, Winer EP, Rimer BK (1993). Why women gain weight with adjuvant chemotherapy for breast cancer. J. Clin. Oncol.

[b33] McTiernan A, Rajan KB, Tworoger SS, Irwin M, Bernstein L, Baumgartner R (2003). Adiposity and sex hormones in postmenopausal breast cancer survivors. J. Clin. Oncol.

[b34] Modugno F, Kip KE, Cochrane B, Kuller L, Klug T, Rohan T (2006). Obesity, hormone therapy, estrogen metabolism and risk of postmenopausal breast cancer. Int. J. Cancer.

[b35] McCullough ML, Feigelson HS, Diver WR, Patel AV, Thun MJ, Calle EE (2005). Risk factors for fatal breast cancer in African-American women and White women in a large US prospective cohort. Am. J. Epidemiol.

[b36] Lu Y, Ma H, Malone KE, Norman S, Sullivan-Halley J, Strom B (2011). Obesity and survival among black women and white women 35 to 64 years of age at diagnosis with invasive breast cancer. J. Clin. Oncol.

[b37] Kwan ML, Chen WY, Kroenke CH, Weltzien E, Beasley J, Nechuta S (2012). Pre-diagnosis body mass index and survival after breast cancer in the After Breast Cancer Pooling Project. Breast Cancer Res. Treat.

[b38] Caan BJ, Kwan ML, Shu XO, Pierce J, Patterson R, Nechuta S (2012). Weight change and survival after breast cancer in the after breast cancer pooling project. Cancer Epidemiol. Biomarkers Prev.

[b39] Chan DS, Vieira AR, Aune D, Bandera E, Greenwood D, McTiernan A (2014). Body mass index and survival in women with breast cancer-systematic literature review and meta-analysis of 82 follow-up studies. Ann. Oncol.

[b40] Garrow JS, Summerbell CD (1995). Meta-analysis: effect of exercise, with or without dieting, on the body composition of overweight subjects. Eur. J. Clin. Nutr.

